# The Bruton tyrosine kinase inhibitor PCI-32765 ameliorates autoimmune arthritis by inhibition of multiple effector cells

**DOI:** 10.1186/ar3400

**Published:** 2011-07-13

**Authors:** Betty Y Chang, Min Mei Huang, Michelle Francesco, Jun Chen, Jeremy Sokolove, Padmaja Magadala, William H Robinson, Joseph J Buggy

**Affiliations:** 1 Pharmacyclics, Inc., Research Department, Sunnyvale CA, 94085-4521, USA; 2 Stanford University School of Medicine, Division of Immunology and Rheumatology, Stanford, CA. 94305; 3 VA Palo Alto Health Care System, 3801 Miranda Avenue, Palo Alto, CA, 94304, USA

## Abstract

**Introduction:**

The aim was to determine the effect of the Bruton tyrosine kinase (Btk)-selective inhibitor PCI-32765, currently in Phase I/II studies in lymphoma trials, in arthritis and immune-complex (IC) based animal models and describe the underlying cellular mechanisms.

**Methods:**

PCI-32765 was administered in a series of murine IC disease models including collagen-induced arthritis (CIA), collagen antibody-induced arthritis (CAIA), reversed passive anaphylactic reaction (RPA), and passive cutaneous anaphylaxis (PCA). Clinical and pathologic features characteristic of each model were examined following treatment. PCI-32765 was then examined in assays using immune cells relevant to the pathogenesis of arthritis, and where Btk is thought to play a functional role. These included proliferation and calcium mobilization in B cells, cytokine and chemokine production in monocytes/macrophages, degranulation of mast cells and its subsequent cytokine/chemokine production.

**Results:**

PCI-32765 dose-dependently and potently reversed arthritic inflammation in a therapeutic CIA model with an ED_50 _of 2.6 mg/kg/day. PCI-32765 also prevented clinical arthritis in CAIA models. In both models, infiltration of monocytes and macrophages into the synovium was completely inhibited and importantly, the bone and cartilage integrity of the joints were preserved. PCI-32765 reduced inflammation in the Arthus and PCA assays. *In vitro*, PCI-32765 inhibited BCR-activated primary B cell proliferation (IC_50 _= 8 nM). Following FcγR stimulation, PCI-32765 inhibited TNFα, IL-1β and IL-6 production in primary monocytes (IC_50 _= 2.6, 0.5, 3.9 nM, respectively). Following FcεRI stimulation of cultured human mast cells, PCI-32765 inhibited release of histamine, PGD_2_, TNF-α, IL-8 and MCP-1.

**Conclusions:**

PCI-32765 is efficacious in CIA, and in IC models that do not depend upon autoantibody production from B cells. Thus PCI-32765 targets not only B lymphocytes but also monocytes, macrophages and mast cells, which are important Btk-expressing effector cells in arthritis.

## Introduction

Rheumatoid arthritis (RA) is a debilitating systemic disease characterized by circulating autoantibodies, synovial inflammation, pannus formation, and cartilage and bone destruction in affected joints. Initiation of the disease involves the systemic dysregulation of T- and B-lymphocytes, which leads to a breach of self-tolerance, resulting in immune responses directed against self-antigens. During the chronic inflammatory phase of the disease, autoantibodies, and immune complexes (ICs) further activate sentinel and effector cells such as neutrophils, monocytes/macrophages, dendritic cells, and mast cells that infiltrate the synovium and release proinflammatory cytokines and matrix metalloproteases, leading to cartilage destruction. Synovial hyperplasia leads to the formation of a pannus that invades the surrounding cartilage and bone, and inflammation enhances the activity of resident osteoclasts leading to bone erosion [1-3].

Bruton tyrosine kinase (Btk) is a Tec-family kinase that is specifically required for B cell activation following engagement of the B cell antigen receptor (BCR) [4]. In the lymphoid lineage, expression of Btk is restricted to B cells and is not found in T or natural killer (NK) cells. Functional null mutations of Btk in humans cause the inherited disease X-linked agammaglobulinemia (XLA), characterized by a lack of peripheral B cells and very low levels of serum immunoglobulin (Ig) (reviewed in [5,6]). In the mouse, point mutation or deletion of Btk causes X-linked immunodeficiency (*xid*), with about 50% fewer conventional B2 B cells, absent B1 B cells, and reduced serum Ig levels [7,8]. As RA is characterized by polyclonal B cell activation giving rise to B cell expansion and the production of autoantibodies, Btk may be a uniquely attractive target for selective B cell inhibition in RA.

Btk is also expressed in specific cells of the myeloid lineage, and evidence suggests that it contributes to immune-complex mediated activation of the FcγR and FcεR signaling pathways [9-11] in monocytes/macrophages, neutrophils, and mast cells. *xid *mice have reduced FcεR-dependent mast cell degranulation [11] and impaired functioning of macrophages [12,13] including TNFα production [14]. *xid *mice have been shown to be resistant to disease manifestations in collagan-induced arthritis (CIA) models [15], and Btk has been shown to be important for autoantibody production in mice [16-18].

We previously described PCI-32765, which is a selective and irreversible inhibitor of Btk [19] that is currently in phase I/II clinical trials in patients with B cell non-Hodgkin lymphoma [20,21]. PCI-32765 blocked BCR signaling selectively in human B cells, but did not affect T cell receptor (TCR) signaling. Inhibition of Btk by PCI-32765 *in vitro *and *in vivo *was monitored using a fluorescent affinity probe for Btk, and inhibition of Btk was tightly correlated with the blockade of BCR signaling and efficacy in disease models. In this report, we investigate the mechanism of action of PCI-32765 in arthritis by studying its effect in *in vivo *models of disease as well as functional studies in primary B lymphocytes, and in monocytes, macrophages, and mast cells. PCI-32765 treatment resulted in potent inhibition of joint synovitis, cartilage, and bone destruction in both CIA and collagen antibody-induced arthritis (CAIA) models, and inhibited inflammation and vasculitis in Arthus and passive cutaneous anaphylactic (PCA) assays. Significant inhibition of BCR-mediated B lymphocyte proliferation and function was observed as expected. However, additionally, inhibition of cytokine release in primary monocytes/macrophages, and inhibition of histamine, prostaglandin (PG) D_2_, TNFα, and IL-8 release from human mast cells was observed following FcγR and FcεR activation. Together, these results argue that Btk inhibition suppresses inflammation, bone erosion, and autoimmunity *in vivo *by affecting the function of multiple immune cells involved in both the propagation and effector phases of CIA.

## Materials and methods

### Drug formulation

For *in vivo *studies, PCI-32765 was formulated in 1% methylcellulose, 0.4% Cremephor^® ^EL (Spectrum Chemicals, Gardena, CA), and 98.09% water.

### Animal studies

All animal studies were designed and conducted under approval from Pharmacyclics Inc. or Bolder BioPath (Boulder, CO, USA) Institutional Animal Care and Use Committee for compliance with regulations prior to study initiation.

### Human samples

Informed consent was obtained from all participating patients from which samples were obtained, and the studies were approved by the Stanford University Institutional Review Board.

### Collagen-induced arthritis model

Male DBA1/1OlaHsd mice were injected on days 0 and 21 with Freunds' Complete Adjuvant (Sigma, St Louis, MO) containing bovine type II collagen. On days 21 to 35, mice were randomized into treatment groups when the average clinical score of each animal was 1.5 (in a scale of 5). Daily drug treatment (PO) was initiated following enrollment and continued for 18 days. Clinical scores were given to each mouse daily for each paw. Clinical score assessment was made using the following criteria: 0 = normal; 1 = one hind paw or fore paw joint affected or minimal diffuse erythema and swelling; 2 = two hind or fore paw joints affected or mild diffuse erythema and swelling; 3 = three hind or fore paw joints affected or moderate diffuse erythema and swelling; 4 = marked diffuse erythema and swelling or four digit joints affected; 5 = severe diffuse erythema and severe swelling of entire paw, unable to flex digits.

### Collagen-antibody induced arthritis model

Male DBA/1 mice were passively sensitized by IV administration of 2 mg of CIA five clone monoclonal antibody blend (Chondrex, Redmond, WA, USA) of IgG2a and IgG2b isotypes on day 0, followed by lipopolysaccharide (LPS) (25 μg) administered IP on day 2 using methods described before [22]. PCI-32765 was administered orally for 14 days starting four hours after antibody challenge on day 0. Assessment of clinical disease was identical to those described of the CIA model.

### Probe assays

Pharmacodynamic probe assay were performed according to Honigberg et al [19] using splenocytes isolated from CIA-treated mice that were sacrificed at three hours or 24 hours following their final dosage of PCI-32765 on day 18.

### Primary human mast cell assays

Human mast cells with phenotypic characteristics of connective tissue-type mast cells or mucosal-type mast cells were derived from buffy coats of healthy human donors prepared as previously described [10,23] and drug studies were performed at Apollonian Biosystems (Palo Alto, CA, USA). As connective-tissue type mast cells were produced at higher yields from human buffy coats, and its release of histamine, PDG_2_, TNF-α, and IL-8 upon IgE/anti-IgE stimulations were comparable, they were used in all the mast cell assays except for the MCP-1 studies. MCP-1 was only released in optimal amounts in the mucosal type mast cells but not the connective-tissue type mast cells and therefore the former was used for the MCP-1 studies. IgE-dependent activation of human mast cells and measurement of histamine levels was assessed as previously described [10]. Mature mast cells were sensitized with human myeloma IgE and treated with PCI-32765 for 10 minutes at 37°C before stimulation with anti-IgE (Sigma, St Louis, MO, USA) or aggregated IgG_1 _(both at 1 mg/mL) [24] at 37°C for 30 minutes for release of histamine and PGD_2_, or with anti-IgE (0.25 mg/mL) for 24 hours for release of TNFα, IL-8, and MCP-1. Histamine content is expressed as a percentage of total histamine release = (histamine in supernatant)/(histamine in supernatant + histamine in cell pellets) × 100%. The amounts of PGD_2_, TNF, IL-8, and MCP-1 in supernatants were determined using PGD_2 _EIA Kit from Cayman (Ann Arbor, MI, USA) and OptEIA Human IL-8, TNF-α, MCP-1 ELISA sets from BD Biosciences (Franklin Lakes, NJ, USA).

### Calcium mobilization assay

Purified B-lymphocytes were washed and resuspended in HBSS and then incubated with 0.5 μM Fura-2, AM (acetoxymethyl ester) (Invitrogen, Carlsbad, CA, USA) at 10^6 ^cells/mL in fresh media at 37°C for 30 minutes. Cells were then treated with vehicle or serial dilutions of PCI-32765 for five minutes and then stimulated with goat anti-human IgM F(ab')_2 _(Invitrogen, Carlsbad, CA) at 10 μg/mL to induce calcium mobilization. THP-1 cells pretreated with IFN-γ at 100 ng/mL for 24 hours or purified human monocytes without pretreatment were loaded with Fura-2, AM at 2 μM for 30 minutes. Cells were then incubated with human IgG (Sigma, St Louis, MO) at 20 μg/mL for 30 minutes on ice. The calcium mobilization was initiated by adding into the cuvette goat anti-human IgG (Sigma, St Louis, MO) to 10 μg/mL. The measurement of the ratio of fluorescence were at Ex/Em = 340/510 nm and 380/510 nm (Luminescence Spectrometer, Aminoco-Bowman Series 2, SLM-AMINCO, Rochester, NY, USA) at room temperature.

### Primary B proliferation assays

Primary human B cells were isolated from peripheral blood mononuclear cell of healthy human volunteers (Stanford Blood Center, Palo Alto, CA) by Ficoll-Hypaque gradient (Amersham Biosciences, Piscataway, NJ) separation followed by negative selection using human Miltenyl human B cell Isolation Kit II. In 0.2 mL RPMI plus 10% FBS, 100,000 B cells were treated with PCI-32765 in triplicate wells or vehicle control in 0.1% DMSO final concentration for 30 minutes at 37°C, 5% CO_2_, then cells were stimulated with 10 μg/mL anti-IgM F(ab')_2_, 5 μg/mL anti-CD3/CD28 as a negative control or 0.5 μg/mL PMA (Phorbal 12-myristate 13-acetate) as a positive control. B cells were stimulated for 72 hours at 37°C, 5% CO_2_. Proliferation was measured with Cell Titer Glo reagent (Promega, Madison, WI) and measured on a luminometer. These results were similar to proliferation assays performed using ^3^H- thymidine incorporation (data not shown).

### Phosphoflow assays

Primary B lymphocytes (isolated as described above) were treated with serial dilutions of PCI-32765 for 30 minutes prior to BCR stimulations with anti-IgM F(ab')_2 _(Invitrogen, Carlsbad, CA) at 10 μg/mL for 10 minutes at 37°C. Also, 3.3 mM hydrogen peroxide was added [25]. Stimulations were terminated by fixing the cells with 1.4% formaldehyde at RT for 15 minutes. Cells were permeabilized and stained according to Irish et al [25]. Cells were stained with anti-CD20-PerCP-Cy5.5 (BD 558021), anti-pBtk (Y551) Alexa647 (BD 558134), pERK1/2 (pT202/204), Pacific Blue (BD 560314), and pPLCγ (Y759)- Alexa488 (BD 558507). Per well, 10,000 events CD20^+ ^events were collected in triplicate.

### Primary human monocyte, human and mouse macrophage assays

Human primary monocytes were isolated from buffy coats of healthy human volunteers (Stanford Blood Bank, Palo Alto, CA, USA) using Ficoll-Paque (GE Healthcare, Piscataway, NJ) and Percoll (GE Healthcare, Pistcataway, NJ) gradients and enriched to 90 to 95% pure CD14^+ ^monocytes with Stemcell EasySep kit for monocyte enrichment using negative selection. Monocytes were subsequently cultured in RPMI plus 10% low-IgG FBS (Invitrogen, Carlsbad, CA) and 100 ng/mL GM-CSF (R&D Systems, Minneapolis, MN) for five to seven days to drive differentiation into macrophages [26]. Murine peritoneal macrophages were enriched by IP injections of 6% thioglycolate [9] into male DBA/one mice for four days. Human primary monocytes/monocyte-derived macrophages or mouse macrophages were stimulated with plate-bound human or mouse IgG (100 μg/mL, Sigma, St Louis, MO), respectively [27]. Per 96-well, 200,000 cells were incubated at 37°C in the presence of PCI-32765, and cell supernatants were harvested at four hours (for TNFα), or 18 hours (for IL-6, IL-1β, MCP-1). TNFα, IL-6, IL-1β, MCP-1 concentrations were measured with ELISA kits from R&D Systems (Minneapolis, MN).

### Citrullinated fibrinogen immune complex-mediated macrophage stimulation

Macrophage stimulation was performed as previously described [28]. Briefly, IC was generated *in vitro *by incubation citrullinated fibrinogen (cFb) with a polyclonal rabbit antibody against human fibrinogen (Dako Cytomation, Carpinteria, CA) at 37°C for 45 minutes. Cross titration of antibody and antigen yielded an optimal ratio for formation of ICs: a final concentration of 10 μg/mL of Fb and 50 μg/mL of antibody were used for IC stimulation of RAW 267.4 cells. At final dilutions, all reagents used in the stimulation assays were tested for endotoxin contamination by the Limulus amebocyte assay (Associates of Cape Cod, Inc., East Falmouth, MA), according to the manufacturer's instructions, and were shown to possess endotoxin levels below the detectable range (< 0.03 EU/mL). For inhibition experiments, plated macrophages were pretreated with inhibitor at concentrations specified for 30 minutes before addition of ICs.

For IC stimulation of human monocyte-derived macrophages, human IgG derived from patients with ACPA (anti-citrullinated protein antibody) positive RA was used to generate plate-bound human cFb-IC. IgG was purified from three pooled plasma samples shown by ELISA to contain high levels of anti-cFb antibodies. IgG was purified by affinity chromatography on protein G columns (Pierce, Rockford, IL), according to the manufacturer's instructions. The eluted IgG fractions were concentrated by centrifugation with buffer exchange to PBS (Amicon ultra, Millipore, Billerica, MA) and were depleted of endotoxin by filtration through a Polymyxin B column (Detoxigel, Pierce, Rockford, IL). IgG concentrations were estimated by optical density (OD) at 280 nm, aliquoted, and stored at -80°C. For generation of cFb-IC, 96-well cell culture plates were coated overnight with 50 μl of cFb (20 μg/mL) at 4°C and washed in PBS containing 0.05% Tween-20 and then incubated for two hours at 4°C with 100 μl of anti-cFb-positive IgG (2.5 mg/mL) or, as a control, with citrullination buffer alone. Wells were again washed in PBS containing 0.05% Tween-20, and macrophages (50 to 75,000/well) were then added to the wells in 200 μl of RMPI containing 5% FCS. For inhibition experiments, monocyte-derived macrophage were gently removed from culture flasks, pretreated with inhibitor at concentrations specified for 30 minutes before addition of pretreated macrophages to plate bound ICs.

### PCA assay

Female Balb/c mice six to seven weeks old were injected intradermally with 500 ng anti-DNP (dinitrophenol) IgE (20 μl). Twenty-one hours later, mice were orally dosed with vehicle, PCI-32765 at 3.125, 6.25, or 12.5 mg/kg. Salbutamol (10 mg/kg) treated mice were dosed 30 minutes prior to challenge, and used as a positive control. Twenty-four hours after sensitization, mice were challenged with 150 μl of DNP-BSA at 1 mg/mL in 1% Evans blue intravenously. Thirty minutes after challenge, mice were sacrificed, and the back skin was removed. The extravasation of Evans blue dye was quantified by measuring the vertical and horizontal diameters of the blue color and multiplication of the two to derive the extravasation area. Three skin punches were removed, placed in extraction buffer (1 N KOH, 1.2 N H_3_PO_4_/Acetone (5:13)) overnight to extract the Evans blue. The concentration of the extravasated Evans blue was measured with SpectraMax (Molecular Devices, Sunnyvale, CA, USA) at OD610.

### Arthus assay

Female Balb/c mice of six to seven weeks were challenged intravenously with 1% ovalbumin (OVA) in saline (10 mg/kg) containing 1% Evans blue dye. Ten minutes later, mice were anesthesized and rabbit anti-OVA injected intradermally on the left side of the back at three adjacent locations. Rabbit polyclonal IgG was injected on the contralateral side as negative control [27]. PCI-32765 or vehicle was administered to animals 60 minutes before the antibody/antigen challenge. Four hours after the challenge, the animals were euthanized, and skin tissue was assessed for edema and inflammation by measuring dye extravasation into the surrounding tissue. Punch biopsies of the injections sites (8 mm) were incubated in 2 mL of formamide overnight at 80°C. The concentration of the extravasated Evans blue was measured with SpectraMax at OD610.

### Micro-CT

Micro-CT analysis was performed on a representative subset of hind/fore limbs excised from CIA (or CAIA) mice treated with vehicle alone or 12.5 mg/kg/day PCI-32765 for 18 (or 14) days. Animal micro-CT scanner used was a GE RS150 small animal micro-CT scanner. The acquisition parameters were: 70 kVp, 25 mA, 20-ms exposure time, 500 views over 200°. Bone pathology was scored from the CT scans using the criteria below. 3D isosurface renderings were generated from the CT scans using commercially available image processing software (Amira^®^, Visage Imaging, San Diego, CA, USA) [29]. All images were reviewed before scoring was initiated to insure that the threshold values were consistent, and to get a preliminary estimate of the range of lesion severity. Each image was then examined across all three dimensions using the aforementioned scoring criteria by a scientist blinded to the study. The J scores were tabulated and analyzed for statistical significance. Lesions in the forelimb or hindlimb were scored with values of 0, 1, 2, 3, or 4 based on a qualitative assessment of lesion size as defined by: 0: Normal bone, 1: Minimal lesions. Some roughening of the isosurface. Small areas of apparent bone resorption. 2: Mild. More numerous lesions. Significant roughening of the isosurface. Full thickness lesions apparent. 3: Moderate. Full thickness lesions larger and more numerous. 4: Marked. Many, large, full thickness lesions. Significant distortion of remaining structure. Marked bone loss.

### Histopathology

Four joints (L/R carpus and tarsus) of the CIA mice and six joints (L/R tarsus, carpus and knee) of the CAIA mice were fixed in phosphate-buffered 10% formaldehyde, decalcified, and each limb was processed in paraffin blocks and sectioned at 5 μm. Sections were stained with H&E and Safranin-O and examined by light microscopy. Scoring (0 to 5) of inflammation, pannus, cartilage, and bone damage was performed by a single blinded pathologist using a modified Mankin score [30,31].

### Synovial fluid cytokine measurements

Synovial fluid of the knee was collected by lavaging the viscous fluid under the patella and around the joints, followed by flushing 2 × with 25 μl of ice-cold PBS, and analyzed using multiplexed immunoassay methods described previously [31]. Cytospin slides containing the infiltrating inflammatory cells were dried overnight, stained with Giemsa-Wright stain and each slide was scored manually, and five fields at 100 × magnification were enumerated for granulocytes, macrophages, and lymphocytes [31].

### Statistics

Analyzes were performed using GraphPad Prism version 4.0 (GraphPad Software, San Diego, CA, USA). Statistically significant differences were determined using one-way analysis of variance with Bonferroni's *post hoc *comparison (for more than three groups) or a two-tailed unpaired Student's *t*-test (for two groups) for all the analyses except for the clinical scores of the CIA and CAIA models where Mann-Whitney U tests and Student t-tests were performed. *p *values less than 0.05 were considered significant.

## Results

### PCI-32765 dose-dependently ameliorates joint inflammation and protects bone and cartilage in collagen-induced arthritis

**Figure 1:**
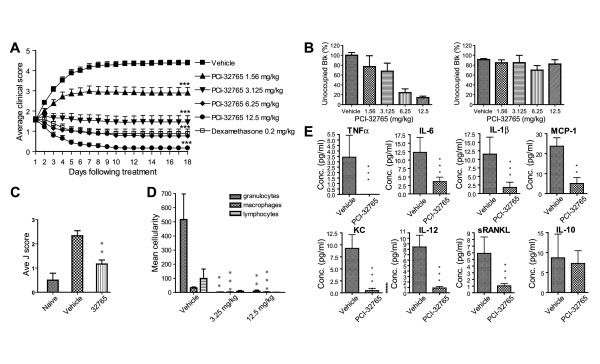
**PCI-32765 dose-dependently inhibits inflammation, bone erosion, cellular infiltration and synovial fluid cytokines/chemokines in CIA models**. **(a) **Mice (*n *= 12) were treated with PCI-32765 orally once daily. Daily average clinical scores are plotted over 18 days of treatment; *** *P *< 0.001 compared with vehicle (Mann-Whitney U-test). **(b) **Using the fluorescent probe PCI-33380 [19], the percentage of Btk occupied by PCI-32765 in collagen-induced arthritis (CIA) mice was measured in splenocytes collected at three hours (left panel, *n *= 6) and 24 hours (right panel, *n *= 6) following last dose (D18) of drug treatment. **(c) **Average J score was determined following micro-CT scanned reconstructed images of hind legs from CIA study (*n *= 6). **(d) **Average cellularity (*n *= 12) in synovial fluid from the knee of CIA mice following PCI-32765 treatment. Giemsa-Wright stained slides were enumerated for granulocytes, macrophages, and lymphocytes under 100×, and five random fields were scored and averaged. **(e) **Synovial fluid cytokine and chemokine levels from the 12.5 mg/kg treatment group (*n *= 12) are shown; * *P *< 0.05, ** *P *< 0.01; *** *P *< 0.001 compared with vehicle, analysis of variance.

**Figure 2:**
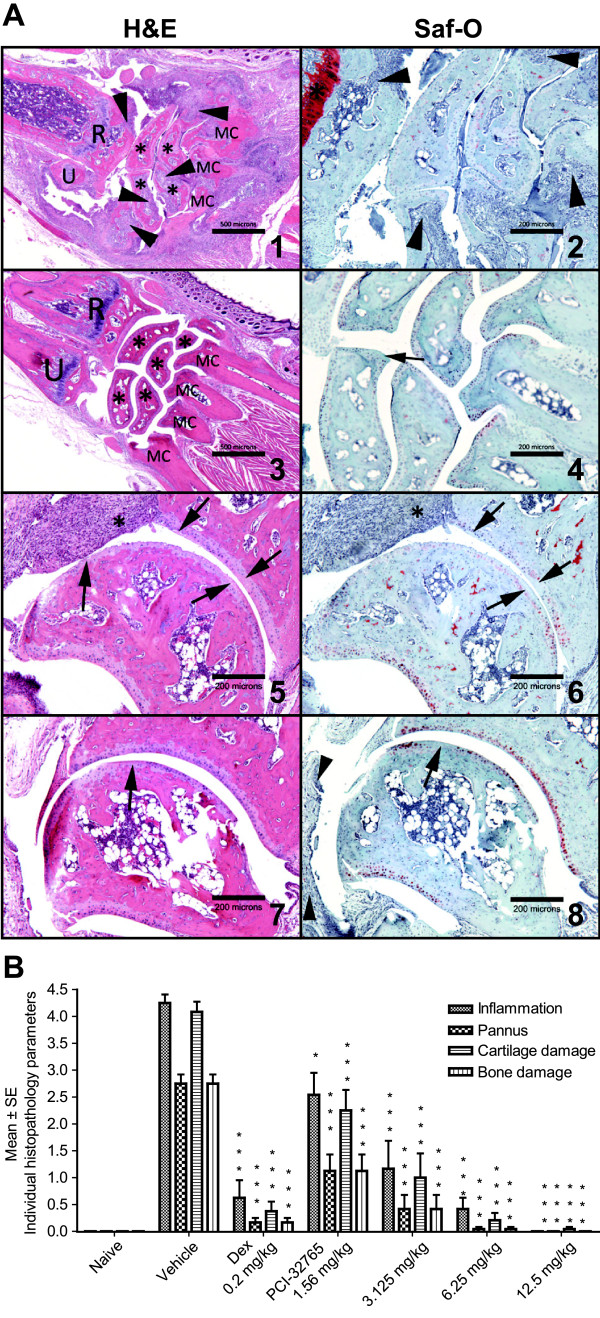
**Histopathology images and scores from CIA mice**. **(a) **Histopathology images from collagen-induced arthritis (CIA) mice: 1, 2, 5, and 6 were treated with vehicle; 3, 4, 7, and 8 were treated with 12.5 mg/kg PCI-32765; 1, 2, 3, and 4 were from carpus, 5, 6, 7, and 8 were from tarsus. Images 1, and 3 are magnified 40 ×; 2, 4, 5, 6, 7, and 8 are magnified 100× under the microscope. Bone resorption is depicted with arrowheads; asterisks show carpal bones in panels 1, and 3. R, radius; U, ulna; MC, metacarpal. In panel 5, and 6 asterisks show severe inflammation, and arrows show loss and necrosis of cartilage. In panels 7, and 8 arrowheads show minimum synovial inflammation, and arrow points to minor loss of articular chondrocytes. **(b) **Histopathology Scores from four joints (carpus and tarsus, both sides) of the CIA mice were averaged and scored by degree of inflammation, pannus, cartilage damage, and bone damage. * *P *< 0.05, ** *P *< 0.01; *** *P *< 0.001 compared with vehicle, analysis of variance.

To study the mechanism of disease suppression in CIA, we treated mice with established disease (average clinical score of 1.5 in a scale of 5) with PCI-32765 at four dose levels (1.56, 3.125, 6.25, and 12.5 mg/kg/day) daily for 18 days. Consistent with previously reported results [19], PCI-32765 dose-dependently and significantly inhibited clinical scores in arthritic mice compared with vehicle-treated mice at all four doses. The ED_50 _in this assay was 2.6 mg/kg. Both the 6.25 mg/kg and 12.5 mg/kg dose levels showed statistically significant disease regression from day 2 to day 18. The 12.5 mg/kg treatment completely reversed arthritic symptoms after 10 days of administration (Figure 1a). A fluorescent probe assay [19] confirmed dose-dependent occupancy of Btk by PCI-32765 in the spleen, with 12.5 mg/kg treatment resulting in 85 to 90% occupancy of Btk measured three hours after the final oral dose, and returning to baseline by 24 hours (Figure 1b). Following 18 days of treatment, PCI-32765-treated mice had no overt signs of toxicity as assayed by changes in body weight gain, complete blood count and clinical chemistry, and the absolute cell counts of B lymphocyte subpopulations (or others) in the spleen were not significantly altered following PCI-32765 treatments [see Table S1 of Additional file 1]. Joints (Left and right paws) of the CIA mice were then examined by histopathology. PCI-32765-treated mice had a marked and dose-dependent reduction in the severity of inflammation, pannus, cartilage destruction, and bone resorption (Figure 2b). The 12.5 mg/kg treated animals had very little inflammation, pannus, cartilage damage, or bone resorption in the carpus and tarsus joint areas where disease is most prominent in CIA (Figure 2a). As histopathology suggested a reduction in bone degradation, we directly measured bone integrity by micro-CT scanning. J scores derived from 3D rendered images revealed a significant reduction in bone damage compared with vehicle controls (Figure 1c). Collectively, these findings demonstrate PCI-32765 reduces the severity of not only inflammation and pannus but also protects CIA mice from bone and cartilage destruction.

To determine the inflammatory cell types affected in the joint, we examined the cellularity of the synovial fluid by scoring for the numbers of granulocyte, macrophage, and lymphocyte infiltration. PCI-32765 treatment drastically reduced the presence of these inflammatory cells in synovial fluid washouts (Figure 1d). Consistent with the reduction of inflammatory cells, cytokines and chemokines such as TNF-α, IL-6, IL-1β, MCP-1, KC, IL-12, and sRANKL from synovial fluid exudates were also significantly reduced in the PCI-32765-treated mice compared with the vehicle control (Figure 1e). These cytokines/chemokines play a central role in the pathogenesis of RA by activating and recruiting monocytes, macrophages, and neutrophils to the inflammatory joints [32]. Interestingly, the levels of IL-10, a Th2 cytokine, were not significantly reduced in the PCI-32765 treated animals. The drug treatments also reduced systemic levels of cytokines IL-6, KC, IL-17 and IFNγ to levels of un-diseased naïve mice [see Figure S1 of Additional file 2].

### PCI-32765 directly affects the function of multiple immune cells *in vitro*

**Figure 3:**
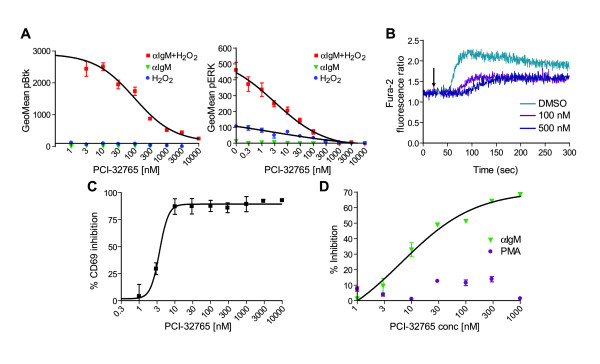
**PCI-32765 potently inhibits anti-IgM stimulation of pBtk and pERK, calcium mobilization, early activation marker and anti-IgM induced cell proliferation in human primary B lymphocytes**. **(a) **Inhibition of intracellular pBtk (Y551) and pERK1/2 staining in B cells following anti-IgM stimulation with flow cytometry methods. **(b) **Inhibition of calcium mobilization following B-cell antigen receptor (BCR) stimulation in primary B cells. **(c) **Inhibition of the early activation marker CD69 following BCR stimulation in primary B cells. **(d) **PCI-32765 inhibits B lymphocyte proliferation stimulated by anti-IgM but not phorbol myristate acetate. Anti-CD3/28 failed to stimulate purified human B lymphocytes (not shown).

To address the mechanism of action of PCI-32765, we wished to determine whether PCI-32765 directly affects the function of immune cells *in vitro*. Btk has a well-defined role in BCR-mediated signaling in B lymphocytes (reviewed in [4]), so we first determined the effects of PCI-32765 on B lymphocyte function *in vitro*. In human primary cells stimulated with anti-IgM, PCI-32765 inhibited tyrosine phosphorylation of Btk at Y551 (IC_50 _= 96.4 nM), and further inhibited phosphorylation of the downstream ERK1/2 with an IC_50 _of 9.5 nM (Figure 3a). Activated Btk phosphorylates PLCγ initiating calcium mobilization. Consistent with this, PCI-32765 dose-dependently inhibited calcium mobilization in primary human B lymphocytes (Figure 3b) as described in the literature correlating Y551 and calcium mobilization following BCR activation [33,34]. Inhibition of B cell activation was also demonstrated by a dose-dependent reduction in the B cell early activation marker CD69 following anti-IgM stimulation with an IC_50 _of 3.7 nM (Figure 3c). PCI-32765 dose-dependently inhibited anti-IgM stimulated B lymphocyte proliferation (IC_50 _= 8 nM) but not proliferation stimulated by PMA, which activates the PKC pathways (Figure 3d). These B lymphocytes did not proliferate in response to anti-CD3/anti-CD28 stimulations as expected (data not shown). Collectively, these results demonstrate that PCI-32765 directly inhibits Btk activity stimulated by BCR activation in primary B lymphocytes.

**Figure 4:**
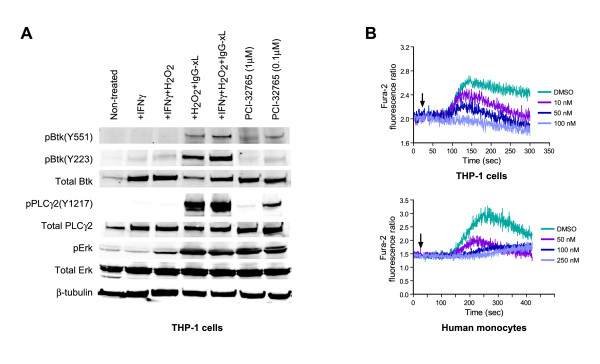
**PCI-32765 inhibits FcγR signaling in monocytes**. **(a) **Inhibition of Btk signaling in THP-1 cells. THP-1 cells were stimulated with IFN-γ and pretreated with PCI-32765 (1 μM or 0.1 μM) or vehicle and stimulated by cross-linking with human IgG and goat anti-human IgG in the presence of H_2_O_2 _and IFNγ. Cell lysates were subjected to western blot analysis and probed with the indicated antibodies. **(b) **Inhibition of calcium mobilization in THP-1 cells (upper panel) or human monocytes (lower panel) stimulated with cross-linked IgG as described in Materials and Methods.

Btk also functions downstream of immune-complex mediated FcγR and FcεR pathways in multiple effector cells [9,11,35,36], therefore we next investigated the effects of PCI-32765 on monocytes, macrophages, and mast cells following Fc receptor stimulation. In the human monocytic cell line THP-1, FcγR crosslinking led to phosphorylation of the Btk autophosphorylation site (Y223) in the SH3 domain, as well as phosphorylation of Btk's physiological substrate PLCγ and the further downstream MAP kinase ERK, consistent with other results [36]. PCI-32765 treatment inhibited Btk autophosphorylation at Y223 as well as phosphorylation of PLCγ, but unlike the case in B cells, did not significantly inhibit levels of phosphorylated ERK (Figure 4a). Consistent with inhibition of PLCγ, PCI-32765 blocked calcium mobilization in THP-1 cells and primary human monocytes (Figure 4b), at similar concentrations as was observed in B cells. In THP-1 cells, using similar stimulatory conditions, PCI-32765 dose-dependently inhibited TNF-α and IL-1β production (IC_50 _= 0.9 nM and 5.2 nM, respectively). However, when THP-1 cells were activated with LPS, PCI-32765 failed to inhibit release of TNF-α at concentrations of up to 10 μM (Table 1), consistent with literature reports suggesting Btk does not play a role downstream of TLR4 signaling [37]. We next determined the effect of PCI-32765 on primary monocytes purified from the peripheral blood of healthy volunteers. PCI-32765 dose-dependently inhibited the release of TNF-α, IL-6, and IL-1β following FcγR cross-linking with IC_50_s of 2.6 nM, 3.9 nM, and 0.5 nM, respectively, and also inhibited release of MCP-1 (Table 1). Next, human monocytes were cultured in the presence of GM-CSF to drive differentiation into macrophages. PCI-32765 inhibited FcγR stimulated release of TNFα, IL-6, IL-1β, and MCP-1 from macrophages as well with IC_50 _of 561, 1299, 26.6, and 422 nM, respectively, albeit less potently as compared with monocytes (Table 1). TNFα, IL-6, and IL-1β release following FcγR stimulation of primary mouse peritoneal macrophages were potently inhibited by PCI-32765 with IC_50_s of 4.9 nM, 0.9 nM, and 5.1 nM, respectively.

**Table 1:** Effect of PCI-32765 on monocytes, macrophages and mast cells

Cell type	Stimulation	**EC**_**50 **_**in nM (peak concentration, pg/mL)**						
		**TNFα**	**IL-6**	**IL-1β**	**MCP-1**	**IL-8**	**Histamine**	**PGD2**

THP-1	FcγR LPS	0.9 (577) > 10,000 (934)	ND > 10,000 (638)	5.2 (80) ND	ND ND			
Primary human monocytes	FcγR LPS	2.6 (6854) > 10,000 (2100)	3.9 (1380) > 10,000 (528)	0.5 (100) ND	908 (3593) ND			
Primary human macrophages Primary mouse macrophages	FcγR FcγR	561 (337) 4.9 (126)	1299 (1500) 0.9 (404)	26.6 (132) 5.1 (22)	422 (1451) 183 (676)			
RAW 267.4 Primary human macrophages	FcγR (cFb) FcγR (cFb)	8.7 (619) 113 (1350)	ND ND	ND ND	ND ND			
Human cultured mast cells	FcεR FcγR	61 (236) 2.1 (67)	ND ND	ND ND	2.2 (341) ND	32 (722) 7.7 (263)	25 (460*) 24 (238*)	21 (1573) 14 (575)
* ng/mL								

PCI-32765 inhibits FcγR-stimulated production of TNFα, IL-6, IL-1β, and MCP-1 in THP-1 cells, human primary monocytes, human macrophages, and mouse macrophages. PCI-32765 inhibits human mast cell releases of histamine, PGD_2_, TNFα, IL-8, and MCP-1 following activations of FcεR and FcγR pathways. THP-1, primary monocytes, and monocyte-derived macrophages were stimulated with high concentration of plate-bound human IgG (or mouse IgG in case of mouse macrophages). RAW 267.4 cells and human macrophages were stimulated with plate-bound citrullinated fibrinogen immune-complexes (ICs) as described in Supplemental Methods [see Additional file 3]. Monocytes and macrophages were pre-treated with PCI-32765 for 30 minutes, and mast cells were treated for 10 minutes prior to stimulations. Mast cells were stimulated with anti-IgE or aggregated IgG_1_. The citrullinated fibrinogen IC experiments were performed twice in triplicates. All other experiments were performed three to five times in triplicate with similar results, and the average IC_50 _of two to three representative experiments is shown. Peak concentrations of readout cytokines/factors are in parenthesis. ND, no data.

In addition, to demonstrate the inhibition of ICs directly implicated in human RA, we stimulated human monocyte-derived macrophages with cFb-IC generated by incubating plate-bound cFb with pooled IgG derived from ACPA-positive RA patients [see Supplementary Methods in Additional file 3]. cFb-IC have previously been demonstrated to mediate macrophage TNF production by co-stimulation of FcγRIIa and TLR4 [28]. PCI-32765 treatment resulted in a dose-dependent inhibition of cFb-IC mediated TNFα production with an IC_50 _of 113 nM (Table 1). This effect is presumably via inhibition of FcγR signaling as experiments with cFb alone or the TLR4 agonist LPS demonstrated no inhibition of TNF production by PCI-32765 (data not shown).

IgE-mediated activation of mast cells occurs through FcεRI receptors and results in degranulation and synthesis of lipid mediators and cytokines [38]. Btk has been reported to function downstream of FcεRI, so we determined the effect of PCI-32765 in human cultured mast cells following crosslinking with anti-IgE antibodies. PCI-32765 potently inhibited histamine and PGD_2 _release following FcεRI activation with an IC_50 _of 25 and 21 nM, respectively (Table 1). Interestingly, LFM-A13, a widely used Btk/JAK2 inhibitor, also inhibited histamine and PGD_2 _release but much less potently (IC_50 _= 14,550 nM and 1,316 nM, respectively). PCI-32765 did not inhibit histamine or PGD_2 _release when triggered by fMLP or ionomycin at concentrations as high as 1 μM, suggesting the effect is mediated by selective inhibition of Btk in mast cells (data not shown). Release of inflammatory cytokines TNF-α, IL-8, and MCP-1 were measured 24 hours following FcεRI activation. PCI-32765 inhibited the release of TNF-α, IL-8, and MCP-1 with IC_50 _of 61 nM, 32 nM, and 2.2 nM, respectively (Table 1). Similarly, these human mast cells were also stimulated with aggregated IgG to activate the FcγR pathways [24], and PCI-32765 also dose-dependently and potently inhibited releases of histamine, PGD_2_, TNFα, and IL-8 with IC_50 _of 24 nM, 14 nM, 2.06 nM, and 7.67 nM, respectively.

Together, these results indicate that PCI-32765 affects multiple immune cells beyond B lymphocytes and suggests that efficacy in CIA may be driven both by inhibiting B cell activation and by inhibiting signaling downstream of FcRs in monocytes, macrophages, and mast cells.

### PCI-32765 dose-dependently ameliorates disease in anti-collagen antibody induced arthritis, reverse passive anaphylaxis assay, and passive cutaneous anaphylaxis models

**Figure 5:**
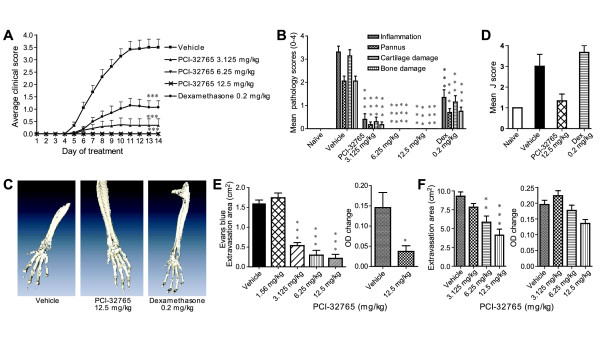
**PCI-32765 dose-dependently inhibits disease in the CAIA, Arthus, and PCA models**. **(a) **Mice (*n *= 10) were treated with PCI-32765 orally once daily. Daily average clinical scores are plotted over 14 days of treatment; ***P < 0.001 (Student t-test). **(b) **Tissue sections from six joints (carpus, tarsus, and knee) of treated mice stained with H&E and Safranin-O were evaluated through histopathology for inflammation, pannus, cartilage damage, and bone erosion (*n *= 10). PCI-32765 treatments significantly inhibited inflammation, pannus, cartilage, and bone damages. **(c) **Representative reconstructed micro-CT images of forelimb of mice treated with vehicle, PCI-32765 (12. 5 mg/kg) or dexamethasone (0.2 mg/kg). **(d) **Mean J scores of bone destruction calculated from micro-CT images (*n *= 3). **(e) **PCI-32765 treatments were evaluated in Arthus reactions induced by OVA and anti-OVA injections. Evans blue dye extravasation area measurements (left) and tissue biopsies evaluated for change of OD (right) are shown (*n *= 6). **(f) **PCI-32765 inhibits anti-DNP (dinitrophenol)-IgE and DNP-BSA mediated skin PCA. Extravasation area (left panel) and change of Evans blue OD (optical density) measurements (*n *= 10) (right panel) are displayed. * *P *< 0.05, ** *P *< 0.01; *** *P *< 0.001 compared with vehicle, analysis of variance. CAIA, collagen antibody-induced arthritis; OVA, ovalbumin; PCA, passive cutaneous anaphylaxis.

We have shown that PCI-32765 directly affects monocyte, macrophage, and mast cell functions *in vitro*, so we wished to determine whether the compound can inhibit acute immune reactions *in vivo *that are driven by these effector cells. To this end, PCI-32765 was evaluated in the mouse CAIA, reversed passive anaphylactic (RPA), and the PCA models. In the CAIA model, anti-collagen antibodies are injected prior to LPS stimulation to induce inflammation. PCI-32765 was administered for 14 days, and clinical scores were monitored daily. Starting at day 5, the majority of animals in the vehicle control group showed inflammation and signs of progressive arthritis. PCI-32765 at 6.25 mg/kg and 12.5 mg/kg treatments led to complete inhibition of arthritic symptoms, whereas the 3.125 mg/kg and dexamethasone treatments led to delayed and significantly reduced arthritic symptoms (Figure 5a). Histopathology of both the left and right paws and knees (six joints) revealed a significant reduction in synovitis, pannus formation, and little to no infiltration of neutrophils, lymphocytes, monocytes, and macrophages into the synovium (Figure 5b). PCI-32765 treatments also protected the bones of the mice, especially in the joint areas (Figure 5c). Micro-CT analysis of forelimbs of the CAIA mice showed a major reduction of J scores measuring bone intensity in the PCI-32765 treated mice compared with vehicle. These differences, however, were not statistically significant, presumably due to the small number of samples tested (*n *= 3). Interestingly, the dexamethasone treatment did not reduce bone loss following treatment although it had a significant improvement in the clinical scores which primarily measure swelling, redness, and inflammation (Figure 5d).

PCI-32765 dose-dependently inhibited IC-mediated acute vasculitis in the RPA assay. The doses of 3.125, 6.25, and 12.5 mg/kg significantly inhibited the Arthus reaction, as assayed by Evans blue dye extravasation area (*P *< 0.001) whereas the lowest dose of 1.56 mg/kg did not (Figure 5e). The total amount of blue dye extracted from skin punches from the 12.5 mg/kg dose group was reduced by 73% (*P *< 0.05) relative to vehicle control.

Finally, we tested the effects of PCI-32765 upon mast cell-dependent PCA reaction following IgE IC activation. Mice were injected intradermally with anti-DNP IgE and treated with PCI-32765, and PCA was elicited three hours later by injection of BSA-DNP and Evans blue dye. PCI-32765 dose-dependently reduced the Evans blue extravasation area, and total Evans blue dye extracted from skin biopsies. At the 6.25 and 12.5 mg/kg dose, Evans blue extravasation area was significantly reduced by 37% and 56%, respectively (*P *< 0.01 and *P *< 0.001, respectively) (Figure 5f). Salbutamol (a short-acting β_2 _receptor agonist) was used as a positive control in this study (data not shown), and was only effective when treated 30 minutes prior to the PCA challenge.

Collectively, these results suggest that PCI-32765 is exerting its effect beyond B lymphocyte function, and on other cell types that express Btk, such as monocytes, macrophages, neutrophils, and mast cells that serve as effector cells following immune-complex mediated inflammation [see Figure S2 in Additional file 4].

## Discussion

Both innate and adaptive immune responses are known to participate in the initiation and progression of RA [1-3]. During the chronic progression of RA, the production of cytokines, chemokines, and metalloproteinases by monocytes, macrophages, and neutrophils lead to the destruction of cartilage and bone. Significant numbers of macrophages, neutrophils, mast cells, and NK cells are present in the RA synovial fluid and tissues. The precise mechanism of how these cells interplay is still under investigation [32,36,39].

The Btk-selective inhibitor PCI-32765 is currently being developed clinically in patients with B cell malignancies and has shown promising activity [20]. Btk is prominently expressed in B cells and has a well-established function in BCR-mediated cell activation and survival [7,40,41]. Btk is also expressed in monocytes, macrophages, neutrophils, and mast cells, and is known to be activated following FcγR/FcεR crosslinking by ICs [9,11,13,36]. Loss of Btk in macrophages and myeloid cells has been linked to compromised inflammatory responses, and Btk-deficient mast cells failed to produce cytokines following FcεRI stimulations. This response was rescued following wild-type Btk but not kinase-dead Btk cDNA transfection [11]. Monocytic and granulocytic cells from Btk deficient *xid *mice and XLA patients are moreover known to be compromised in their responses to FcγR crosslinking and inflammation [13,42]. These observations suggest Btk is an ideal intracellular target for therapeutic intervention in inflammatory and immune-mediated disorders.

We have shown that PCI-32765 potently and dose-dependently reversed clinical arthritis and prevented cartilage and bone erosion in an aggressive late-stage CIA model, and in a CAIA model. In both models, anti-collagen antibodies form ICs that engage and activate the FcγR receptors in monocytes, macrophages, neutrophils, and mast cells, resulting in infiltration/trafficking of these effector cells to antigen sites, such as the joints, where the cells release mediators that initiate synovitis, bone erosion, and pain [22,43,44]. Synovial and articular inflammation is decreased in mice with FcγR deficiency [43,45] demonstrating the importance of FcγR mediated signaling in these cells. In addition, PCI-32765 potently inhibited immune complex and FcγR-mediated acute vasculitis model in mice wherein neutrophils, macrophages, and mast cells, independently of complement, are the key effector cells [46]. These studies collectively establish the suppressive effect of PCI-32765 on IC-mediated inflammation *in vivo*.

Our *in vitro *studies demonstrate that PCI-32765 not only suppresses BCR-mediated B lymphocyte function but also inhibits cytokine/chemokine release upon FcγR activation of monocytes and macrophages, as well as FcεR activation of mast cells. The signal transduction pathway that results in IC-mediated release of cytokines/chemokines from monocytes and macrophages entails Btk activation which is necessary for PLC recruitment and activation, and further downstream signaling, typically through ERK (extracellular signal-regulated kinase), JNK (c-Jun N-terminus kinase), and MAPK (mitogen-activated protein kinase). Consistent with these observations, phosphorylation of Btk and PLCγ and calcium mobilization of FcγR crosslinked monocytes and macrophages were dose-dependently inhibited with PCI-32765, as was TNF-α, IL-6, and IL-1β release. The IC_50_s for cytokine release were in the range of 1.0 to 10 nM in THP-1 cells and primary human monocytes, but were much higher in human macrophages. These results may be related to the fully differentiated nature of the macrophages possessing a higher expression of surface FcγRs (> 10 fold) compared with monocytes or monocytic leukemic cell lines rather than levels of Btk protein expression which are comparable (data not shown). We further directly demonstrated the correlation of inhibition of Btk activity to the reduction of cytokine and chemokine release following FcγR activation because PCI-32765 treatment inhibited phosphorylation of Y223 in Btk and Y1217 in PLCγ in monocytes and THP-1 cells. Interestingly, ERK phosphorylation in these cells was not affected by PCI-32765. This is consistent with some reports suggesting that JNK and p38 phosphorylation, rather than ERK, are the key downstream transducers in FcγR-activated signaling of monocytes/macrophages [47]. The lack of inhibition of LPS-induced cytokine release of TNFα, or IL-6 (both protein or transcripts, data not shown) by PCI-32765 at concentrations up to 10 μM, is consistent with the observations of Perez de Diego et al that Btk is not essential in TLR4-mediated stimulation of monocytes or macrophages [37].

In mast cells cultured from human mononuclear cells, PCI-32765 potently inhibits histamine release as well as lipid mediators (such as PGD_2_) and cytokine/chemokine at equal potency with IC_50_s of about 20 to 60 nM. Previously, it has been shown that mast cells from *xid *and btk null mice exhibit severe impairments in cytokine release but only mild defects in degranulation upon FcεR1 crosslinking [11]. The human peripheral blood-derived mast cells used here may have significant differences with murine bone marrow-derived mast cells used elsewhere, with the former more closely resembling mast cells from lung as opposed to those found in skin [10]. Inhibition of Lyn by continuous exposure to PCI-32765 as might occur under the current culture conditions [19] and may have added to the potency of the suppression of FcεRI degranulations.

In both CIA treatment models and CAIA models, PCI-32765 inhibited clinical inflammation, pannus, and protected against cartilage and bone damage. Mechanistically, PCI-32765 inhibited FcγR-induced cytokines such as TNFα, IL-6, IL-1β, and MCP-1 potently in monocytes albeit less so in macrophages. Additional benefit in this model might have been derived from inhibition of RANKL-induced osteoclastic differentiation as suggested by the findings by Shinohara et al. that Btk and Tec are key downstream elements in RANK signaling [48]. Following PCI-32765 treatments, there was a nearly complete inhibition of infiltrating cells (neutrophils and macrophages) in the synovial joints of mice. In the synovial fluid, cytokines and chemokines such as IL-1β, IL-6, TNFα, and MCP-1 were potently suppressed, consistent with our *in vitro *results with primary monocytes and macrophages [see Figure S2 in Additional file 4].

In combination with the *in vitro *studies with human primary cells, these results suggest that the effects of PCI-32765, a potent Btk inhibitor with demonstrated activity in NHL (non-Hodgkin's lymphoma) patients, has effects on multiple cell types that contribute to the pathogenesis of RA, and is highly effective in several inflammatory disease models including CIA, CAIA, RPA, and PCA. Btk inhibition by PCI-32765 or related molecules is thus a promising direction for therapeutic trials in human IC-mediated diseases such as RA, systemic lupus erythematosus, idiopathic thrombocytopenic purpura, glomerulonephritis, autoimmune-mediated hemolytic anemia, and IC-mediated vasculitis as well as others.

## Conclusions

PCI-32765 is a selective Btk inhibitor that is effective in lymphocyte-independent IC models, such as CAIA, Arthus, and PCA reactions. In vitro, PCI-32765 not only targets BCR-mediated B lymphocyte pathways but also inhibits FcγR-induced release of cytokines such as TNFα, IL-1β, and IL-6 from monocytes, macrophages, and FcεR-induced degranulation of mast cells. Therefore, PCI-32765 targets not only B lymphocytes but also monocytes, macrophages, and mast cells, which are important effector cells in autoimmune arthritis.

## Abbreviations

BCR: B-cell antigen receptor; Btk: Bruton tyrosine kinase; CIA: collagen-induced arthritis; CAIA: collagen antibody-induced arthritis; cFb: citrullinated fibrinogen; ELISA: enzyme-linked immunosorbent assay; FBS: fetal bovine serum; IC: immune complex; NK: natural killer; Ig: immunoglobulin; IL: interleukin; LPS: lipopolysaccharide; OD: optical density; OVA: ovalbumin; PCA: passive cutaneous anaphylactic assay; PG: prostaglandin; RA: rheumatoid arthritis; RPA: reversed passive anaphylactic assay; TCR: T cell receptor; TNF: tumor necrosis factor; xid: X-linked immunodeficiency; XLA: X-linked agammaglobulinemia.

## Competing interests

Dr. Chang, Dr. Huang, Ms. Francesco, Dr. Chen, Ms. Magadala, and Dr. Buggy are employees of Pharmacyclics, and hold stock and/or stock options at Pharmacyclics Inc. Pharmacyclics Inc. owns patents and patent applications covering various aspects of and relating to PCI-32765. Dr. Robinson received research funding from Pharmacyclics for this study. Dr. Sokolove declares no competing interests.

## Authors' contributions

BYC designed, conceived the study, coordinated the project, analyzed the data, performed statistical analysis, and wrote the manuscript. MMH, MF, PM, JC, and JS performed the experiments, analyzed the data, and contributed to the manuscript. JS, and WHR co-designed experiments and contributed to the manuscript. JJB revised the manuscript, managed the project and contributed to the manuscript. All authors read and approved the final manuscript.

## Supplementary Material

Additional file 1 **Table S1**. Immunophenotyping of splenocyte subpopulations following 18 days of treatment with PCI-32765. Click here for file

Additional file 2 **Figure S1**. Serum cytokines/chemokines from collagen-induced arthritis (CIA) mice treated with PCI-32765 at 12.5 mg/kg (*n *= 12) for 18 days. * *P *< 0.05 compared with vehicle, analysis of variance. Click here for file

Additional file 3 **Supplementary materials and methods**. Immunophenotyping of mouse spleens from collagen-induced arthritis (CIA) models. Click here for file

Additional file 4 **Figure S2**. PCI-32765 potentially inhibits multiple pathways in the pathogenesis of rheumatoid arthritis. PCI-32765 inhibits B cell activation, and suppresses cytokine/chemokine production from monocytes, macrophages, and mast cells following immune-complex activation (modified from [49]). Art by Jacqueline Schaffer, M.A.M.S., medical illustrator, for Pharmacyclics Inc. Click here for file
