# Supplementary material for: The Bruton tyrosine kinase inhibitor PCI-32765 ameliorates autoimmune arthritis by inhibition of multiple effector cells
Source: Arthritis Res Ther. 2011 Jul 13;13(4):R115. doi: 10.1186/ar3400 (PMC3239353; doi:10.1186/ar3400)
Supplement: Additional file 1 — Table S1. Immunophenotyping of splenocyte subpopulations following 18 days of treatment with PCI-32765. [file ar3400-S1.DOC]

**Supplementary Table 1.** Absolute numbers of splenic subpopulations following PCI-32765 treatments*

| **Population** | **Naïve**  **(n=4)** | **Vehicle**  **(n=12)** | **PCI-32765**  **3.12 mg/kg**  **(n=12)** | **PCI-32765**  **12.5 mg/kg**  **(n=12)** |
| --- | --- | --- | --- | --- |

| **Total Splenocytes**  **CD4^+^** | 48 ± 9.6  14.6 ± 2.8 | 77 ± 14.5  34.3 ± 4.4 | 84 ± 17  33.5 ± 18.6 | 71 ± 19  26.7 ± 8.2 |
| --- | --- | --- | --- | --- |
| **CD8^+^** | 2.9 ± 0.7 | 5 ± 1.4 | 4 ± 1.4 | 3.6 ± 1.7 |
| **B220^+^** | 23.9 ± 4 | 31.8 ± 13.4 | 38.8 ± 26.7 | 33.3 ± 14.2 |
| **B1** | 9.5 ± 1.8 | 7.6 ± 3 | 10.6 ± 4.9 | 9.8 ± 4.4 |
| **B1a** | 0.08 ± 0.14 | 0.01 ± 0.03 | 0.01 ± 0.02 | 0.03 ± 0.03 |
| **B1b** | 0.06 ± 0.1 | 0.03 ± 0.05 | 0.1 ± 0.17 | 0.16 ± 0.19 |
| **B2** | 11.5 ± 2.3 | 8.7 ± 7.9 | 11.2 ± 8.9 | 13.1 ± 7.6 |
| **MZ** | 7.3 ± 1.3 | 6.8 ± 3 | 9.5 ± 4.6 | 8.7 ± 4.1 |
| **T1** | 0.54 ± 0.07 | 0.44 ± 0.12 | 0.66 ± 0.2 | 0.66 ± 0.26 |
| **CD11b^+^** | 0.8 ± 0.1 | 3.7 ± 1.7 | 2.8 ± 3.4 | 2 ± 2 |

* Splenocytes from naïve or CIA mice treated with vehicle or PCI-32765 for 18 days were stained with specific antibodies and gated for the various subpopulations. Absolute numbers were calculated using frequencies (%) and total cells/spleen for each animal and are expressed as the group mean ± SEM absolute count x 10^6^ cells/spleen. B1 cells are IgM^hi^IgD^lo^, B1a cells are IgM^hi^IgD^lo^ CD5^+^ B220^lo^, B1b cells are IgM^hi^IgD^lo^CD5^-^B220^+^, B2 cells are IgM^lo^IgD^hi^, MZ cells are IgM^hi^IgD^-^CD21^hi^CD23^-^, T1 cells are IgM^+^IgD^-^CD21^-^CD23^+^. This experiment was performed twice with similar results. These numbers are from one representative animal experiment.
