# Supplementary figures and images for: The Bruton tyrosine kinase inhibitor PCI-32765 ameliorates autoimmune arthritis by inhibition of multiple effector cells
Source: Arthritis Res Ther. 2011 Jul 13;13(4):R115. doi: 10.1186/ar3400 (PMC3239353; doi:10.1186/ar3400)

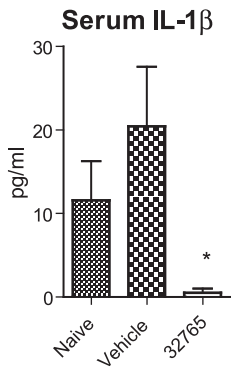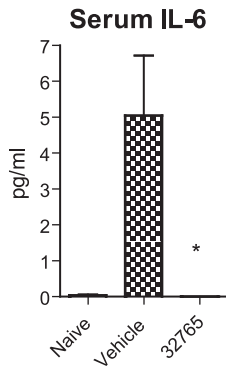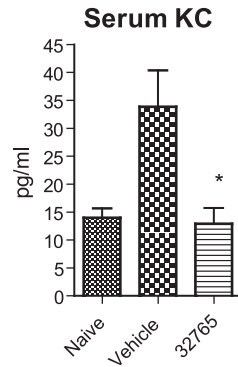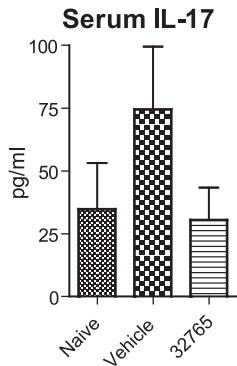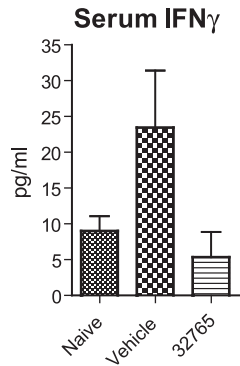

Supplement: Additional file 2 — Figure S1. Serum cytokines/chemokines from collagen-induced arthritis (CIA) mice treated with PCI-32765 at 12.5 mg/kg (n = 12) for 18 days. * P < 0.05 compared with vehicle, analysis of variance. [file ar3400-S2.PDF]

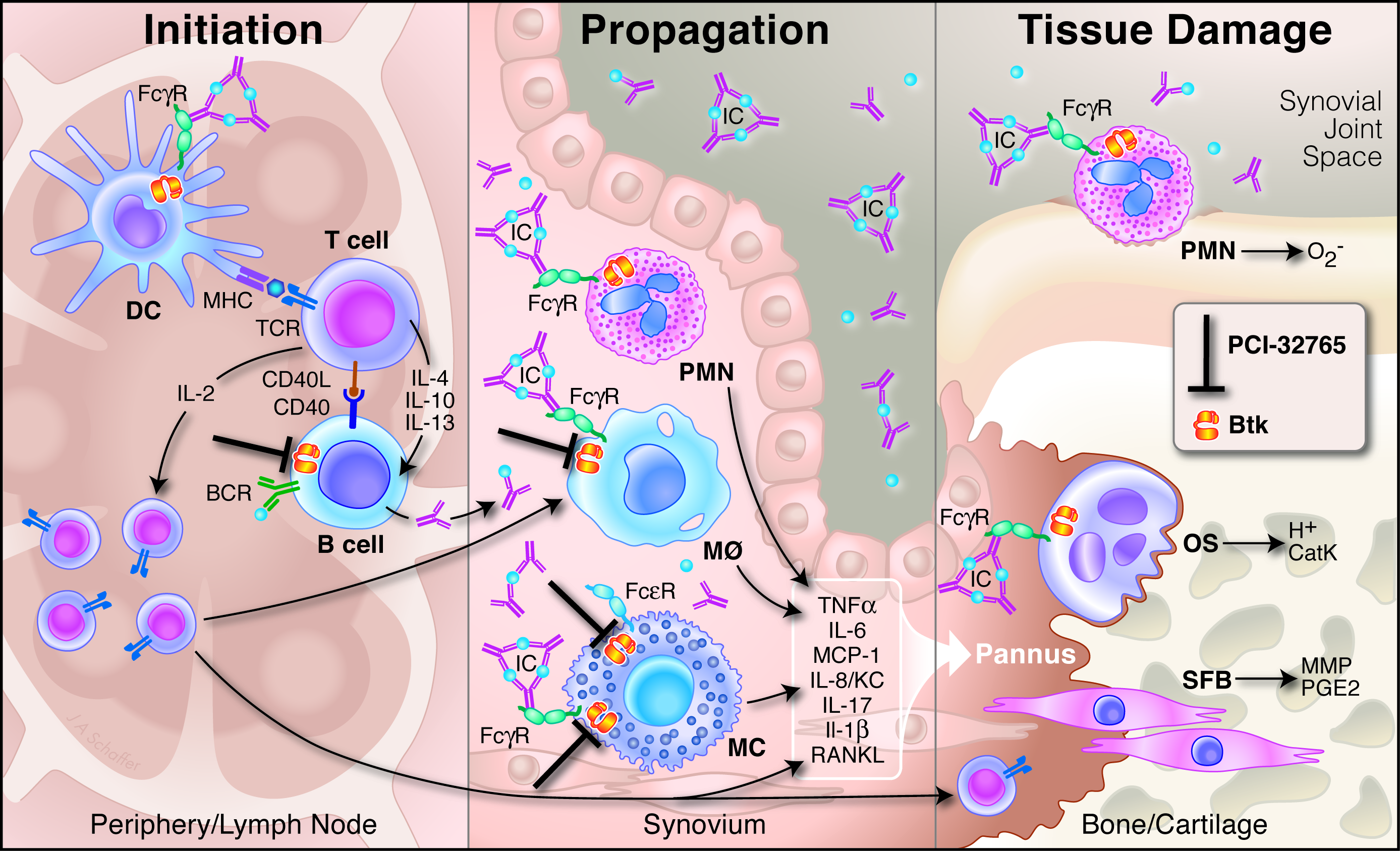

Supplement: Additional file 4 — Figure S2. PCI-32765 potentially inhibits multiple pathways in the pathogenesis of rheumatoid arthritis. PCI-32765 inhibits B cell activation, and suppresses cytokine/chemokine production from monocytes, macrophages, and mast cells following immune-complex activation (modified from [49]). Art by Jacqueline Schaffer, M.A.M.S., medical illustrator, for Pharmacyclics Inc. [file ar3400-S4.TIFF]
