# Supplementary material for: The Bruton tyrosine kinase inhibitor PCI-32765 ameliorates autoimmune arthritis by inhibition of multiple effector cells
Source: Arthritis Res Ther. 2011 Jul 13;13(4):R115. doi: 10.1186/ar3400 (PMC3239353; doi:10.1186/ar3400)
Supplement: Additional file 3 — Supplementary materials and methods. Immunophenotyping of mouse spleens from collagen-induced arthritis (CIA) models. [file ar3400-S3.DOCX]

**Immunophenotyping of mouse spleens from CIA models**

Mouse splenocytes were harvested by passing through a 70μm cell straining with a syringe plunger. The cells were pelleted by centrifugation at 1500 rpm, 5 minutes and incubated in Cell Lysis Buffer and stopped by addition of 5 mL PBS + 5% FBS. The splenocytes were pelleted again by centrifugation, washed with PBS + 5% FBS and pelleted again. The cells were resuspended in 5 mL PBS + 2% FBS. The splenocytes were counted using Countbright Absolute Counting Beads (Molecular Probes). 100 μl cells from each sample were aliquoted into 8 wells of a 96 well plate. 100 μl of antibody cocktail was added to the cells. Staining was done for 1 hour at 4°C then the cells were fixed with 1.4% final concentration of paraformaldehyde. The following antibody panels were used for B cell subsets (B220, CD5, IgD, IgM, CD21, CD23), monocytes (CD16, CD11b, CD45R) and T cells (CD4, CD8, CD44, CD62L). All antibodies were purchased from BD Biosciences (San Jose, CA).
